# New *In Vitro* Interaction-Parasite Reduction Ratio Assay for Early Derisk in Clinical Development of Antimalarial Combinations

**DOI:** 10.1128/aac.00556-22

**Published:** 2022-10-05

**Authors:** Sebastian G. Wicha, Annabelle Walz, Mohammed H. Cherkaoui-Rbati, Nils Bundgaard, Karsten Kuritz, Christin Gumpp, Nathalie Gobeau, Jörg Möhrle, Matthias Rottmann, Claudia Demarta-Gatsi

**Affiliations:** a Department of Clinical Pharmacy, Institute of Pharmacy, University of Hamburg, Hamburg, Germany; b Department of Medical Parasitology and Infection Biology, Swiss Tropical and Public Health Institute, Allschwil, Switzerland; c University of Basel, Basel, Switzerland; d Medicines for Malaria Venture, Geneva, Switzerland; e IntiQuan GmbH, Basel, Switzerland

**Keywords:** *in vitro-in silico* preclinical strategy, drug combinations, drug-drug interactions, pharmacometric models, *Plasmodium*

## Abstract

The development and spread of drug-resistant phenotypes substantially threaten malaria control efforts. Combination therapies have the potential to minimize the risk of resistance development but require intensive preclinical studies to determine optimal combination and dosing regimens. To support the selection of new combinations, we developed a novel *in vitro-in silico* combination approach to help identify the pharmacodynamic interactions of the two antimalarial drugs in a combination which can be plugged into a pharmacokinetic/pharmacodynamic model built with human monotherapy parasitological data to predict the parasitological endpoints of the combination. This makes it possible to optimally select drug combinations and doses for the clinical development of antimalarials. With this assay, we successfully predicted the endpoints of two phase 2 clinical trials in patients with the artefenomel-piperaquine and artefenomel-ferroquine drug combinations. In addition, the predictive performance of our novel *in vitro* model was equivalent to that of the humanized mouse model outcome. Last, our more informative *in vitro* combination assay provided additional insights into the pharmacodynamic drug interactions compared to the *in vivo* systems, e.g., a concentration-dependent change in the maximum killing effect (*E*_max_) and the concentration producing 50% of the killing maximum effect (EC_50_) of piperaquine or artefenomel or a directional reduction of the EC_50_ of ferroquine by artefenomel and a directional reduction of *E*_max_ of ferroquine by artefenomel. Overall, this novel *in vitro-in silico*-based technology will significantly improve and streamline the economic development of new drug combinations for malaria and potentially also in other therapeutic areas.

## INTRODUCTION

Poverty-associated infectious diseases such as malaria still inflict extensive morbidity and mortality in resource-poor countries. In 2020 there were an estimated 241 million malaria cases and 627,000 malaria-related deaths worldwide (1). Malaria eradication poses significant intertwined challenges at the logistical, political, and scientific levels. The World Health Organization (WHO) recommends the development of combination therapies (2) to treat malaria, so that the substances in the combination can rescue each other if resistance to one of them evolves and, together, still provide an adequate clinical and parasitological response (ACPR) of 95%. Given the substantial numbers of possible combinations, the determination of optimal combination and dosing regimens to achieve the target efficacy is complex and requires sophisticated preclinical assays.

Although several new antimalarial combinations have been developed, many of them were discontinued in the clinical phase after efficacy studies in field settings (3, 4), thus stressing the need for more sophisticated preclinical assays to increase the success rate of novel antimalarial combinations in clinical development. This would make it possible to focus only on drug combinations that meet all the criteria and thus accelerate the development and commercialization of new antimalarial combinations. Conventionally, antimalarial monotherapies and recently also antimalarial combinations are evaluated *in vivo* in a severe immunodeficient NSG (NOD/SCID/IL2Rγ^−/−^) mouse model engrafted with human erythrocytes and infected with an adapted Plasmodium falciparum 3D7 strain (*Pfalc*HuEry mouse model) (5–7). Another, even more resource-intensive setting is the human volunteer infection study (VIS), in which healthy volunteers are infected with P. falciparum 3D7 and treated before becoming symptomatic (8). Apart from ethical concerns regarding the use of animals and the considerable amount of time and cost required by these *in vivo* studies, only a small number of doses can be tested, preventing the exploration of the full combined response surface of the antimalarial effects. Another important limitation of these studies is the uncertainty of parasite killing following drug treatment, since conventional methods cannot reliably distinguish viable from dying or dead parasites (9, 10). Indeed, the parasite clearance rate (viable versus dead parasites), measured immediately after treatment, may differ between *in vivo* models owing to different clearance mechanisms of dead parasites from the bloodstream. These inaccuracies make it difficult to estimate the maximum effect of the antimalarials and blur pharmacokinetic (PK)/pharmacodynamic (PD) calculations.

Historically, *in vitro* drug combination assays (11, 12), presented graphically as isobolograms, have been used to evaluate PD drug-drug interactions (DDIs) to inform the development of novel antimalarial combination treatments. These assays aim to determine the drug concentration added to an *in vitro* culture of parasites that reduces their density to 50% of that of the untreated control, called the 50% inhibitory concentrations (IC_50_). The assessment is made at a single time point, usually 72 h after the drug has been added to the culture of parasites. For a combination, an isobole showing the fractional inhibitory concentration (FIC) (the ratio of IC_50_ of the combination to the IC_50_ of the monotherapy) for drug A versus the FIC for drug B is plotted. The PD DDI is then classified as additive, synergistic, or antagonistic if the shape of the isobole is linear, concave, or convex, respectively. However, these assays are static and do not allow identification of the PK/PD properties of a drug, such as the maximum killing effect (*E*_max_) and the concentration producing 50% of the killing maximum effect (EC_50_) in combination. In addition, isobologram approach results are prone to inconsistencies between individual studies. For example, the interaction between tafenoquine and chloroquine was found to be antagonistic or additive by Gorka et al. (13), whereas Bray et al. found the interaction to be synergistic (14). Although these different results may be due to the use of different parasite strains or to the time of drug incubation and subsequent analysis, the discrepancy provides an indication of the limits of the assay. In addition, it is difficult to relate an antagonistic or synergistic effect from an isobologram to the parasitological endpoints. Hence, no current method fully meets the requirement for testing new antimalarial combinations in a reasonable time frame against their chance of success to achieve the ACPR28 target of 95% in field clinical trial patients.

**FIG 1:**
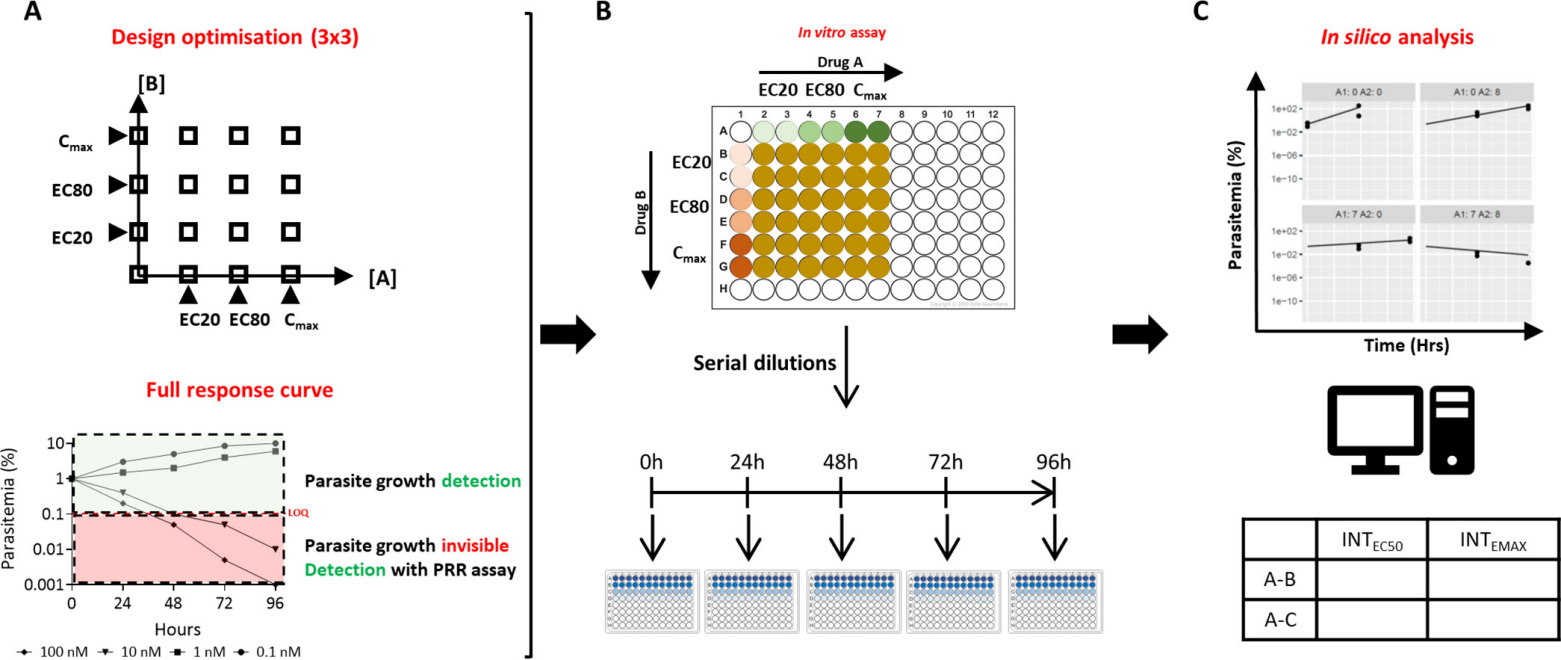
Scheme showing the *in vitro* parasite viability assay for identification of pharmacodynamic antimalarial drug-drug interaction parameters. (A) Schematization of the optimizations made to the assay to provide the relevant data for modeling. The use of effective concentrations (EC_20_, EC_80_, and maximum concentration in serum [*C*_max_]) reduces the number of experiments needed. The PRR assay elucidates the full response curve and can also detect differences and interactions with respect to the rate of parasite killing. (B) Parasite killing kinetics were determined by culturing P. falciparum parasites in the presence of antimalarial drugs at different concentrations (EC_20_, EC_80_, and clinical *C*_max_) as either monotherapy or combinations at different time points. Aliquots corresponding to 1 mL of culture are taken at specific time points and washed, and the free parasites are cultured with fresh erythrocytes under limiting serial dilution conditions. Parasite growth is subsequently monitored after 21 days, and the initial number of viable parasites in the aliquot is calculated (see Materials and Methods). (C) Parasite kinetics were modeled with systems of differential equations. Pharmacodynamic drug interactions were quantified using the GPDI model as shifts of EC_50_ or *E*_max_. The *in vitro* model parameters were used for clinical trial simulations and compared to real phase 2 field clinical trial data.

To overcome these problems, and to optimally select drug combinations and leverage preclinical data to inform first-in-human clinical studies about potential pharmacodynamic DDIs, we have developed a novel *in vitro-in silico-*based combination technology: the interaction-parasite reduction ratio (PRR) assay combined with a PK/PD model-based approach (Fig. 1). This assay is in accordance with the “3Rs” principle by replacing and/or reducing the use of animals, and it aims to minimize the knowledge gap in translational research and clinical development. It is based on the dynamic assay developed for testing antibiotic combinations (15) with rationally selected drug concentrations (16) and on the *Plasmodium* growth inhibition assay in combination with a PRR assay. In contrast to simple growth inhibition, the PRR assay is the gold standard to evaluate the maximum parasite killing and informs about parasite viability at different time points after drug exposure (17). Together, this makes it possible to investigate the parasite reduction ratio of drug combinations at different concentrations. The *in vitro* parasite viability data generated with this assay were used in conjunction with state-of-the art PK/PD modeling techniques to describe the killing rate of drugs over time alone and in combination.

Here, we show how the assay allowed us to identify the PK/PD relationship as a function of the concentrations of drug A and drug B. We describe the development of this novel approach and its application to two new antimalarial drug combinations, recently evaluated in clinical trials, i.e., artefenomel (AF)-piperaquine (PPQ) and AF-ferroquine (FQ). Clinical trial simulations with the *in vitro-in silico* interaction-PRR-derived PK/PD relationship were performed, and predictions were compared with the observations in field clinical trials patients, demonstrating that this hybrid clinical PK-*in vitro* PD interaction model can be exploited for clinical trial simulations of antimalarial drug combinations.

## RESULTS

### The *in vitro* interaction-PRR assay provides informative PD data to successfully quantify complex PD interactions of *E*_max_ and EC_50_.

**(i) AF-PPQ combination.** For the *in vitro* interaction-PRR assay, concentrations of 0, 7, 12 and 100 nM or 0, 8, 12, and 100 nM were selected for AF and PPQ, based on the parasite growth-inhibitory assay pretest and covering the EC_20_ (i.e., the concentration stimulating 20% of the of the maximum effect), the EC_80_, and a “high” concentration covering the clinically relevant maximum concentrations, adapted from the work of Chen et al. (16). If concentrations were chosen suboptimally in a first iteration, more concentration levels were added in a subsequent experiment so that EC_20_, EC_80_, and the maximum effect were covered.

**FIG 2:**
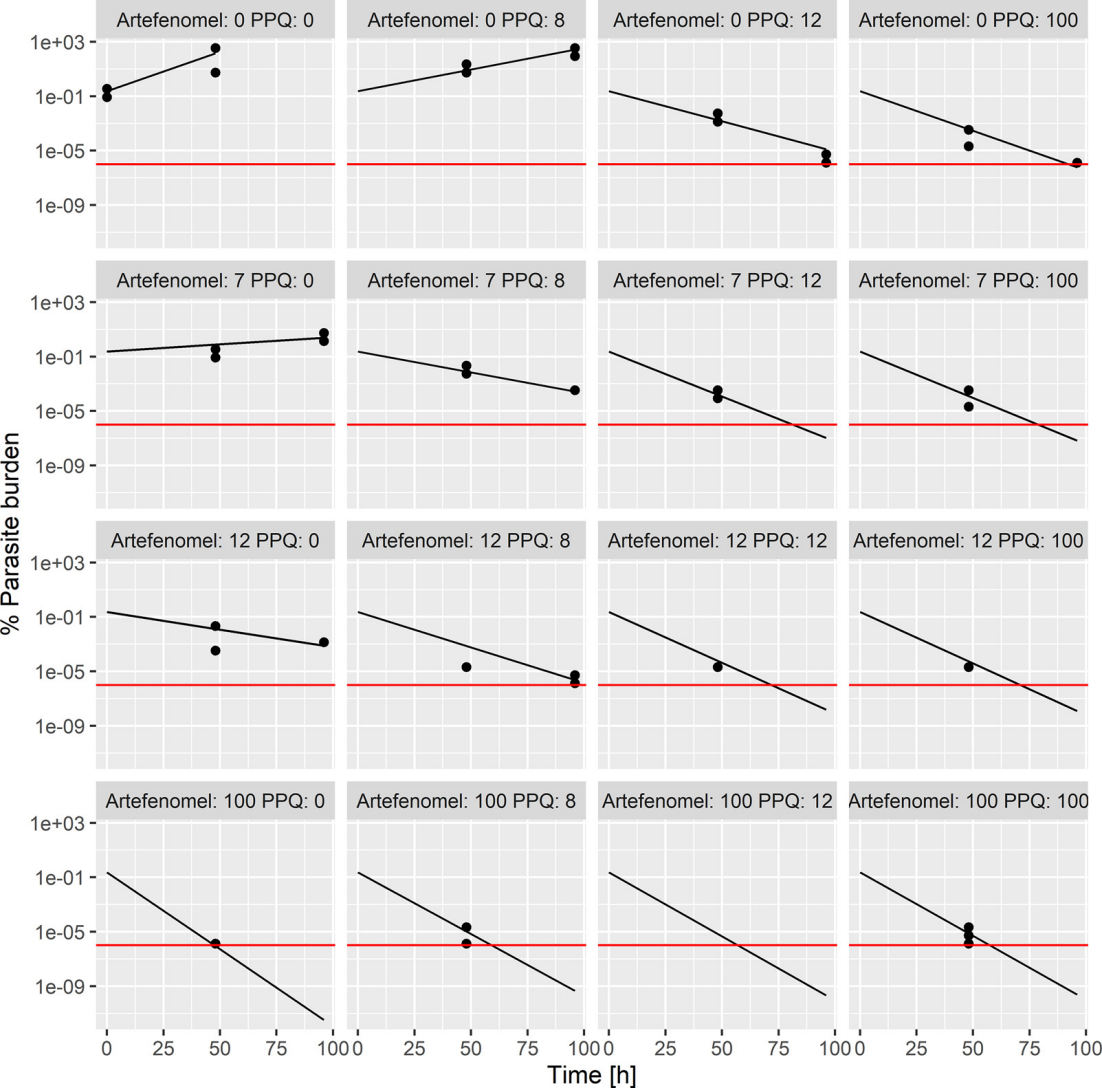
Individual model fits for AF-PPQ for the *in vitro* interaction-PRR assay data. Observed parasite burden (black points) and model-predicted parasite burden (black lines) time courses at different concentrations of AF and PPQ (numbers denote the concentrations [in nanomolar units] of each agent, covering the EC_20_, EC_80_, and a concentration at the maximum effect). Red lines show quantification limits.

The viable-parasite–time course profiles for the AF-PPQ combination were modeled using a time-kill modeling approach based on the *in vitro* interaction-PRR interaction assay data (Fig. 2). Thereby, the combined drug effects were modeled using the general pharmacodynamic interaction (GPDI) model (18), which provided a flexible framework to quantify PD interactions as shifts of potency (EC_50_) or maximum effect (*E*_max_) or both at the same time. Moreover, with the GPDI model, the directionality of the drug interactions, i.e., which drug took the role of the perpetrator or victim, was quantified. The parameter estimates of the PK/PD model are presented in Table S1 in the supplemental material. Assuming Bliss independence as the underlying additivity criterion, the following PD interactions were estimated quantifying deviations from Bliss independence: (i) the EC_50_ of AF was increased by 1,070%, mediated by PPQ; (ii) the EC_50_ of PPQ was reduced by 35.5%, mediated by AF; (iii) the *E*_max_ of AF was reduced by 14.0%, mediated by PPQ; and (iv) the *E*_max_ of PPQ was increased by 31.6%, mediated by AF. Hence, an asymmetric interaction was quantified for the AF-PPQ combination, as both increases and decreases of the PD parameters EC_50_ and *E*_max_ were observed. The model predictions agreed well with the observed parasite burden (Fig. 2).

**FIG 3:**
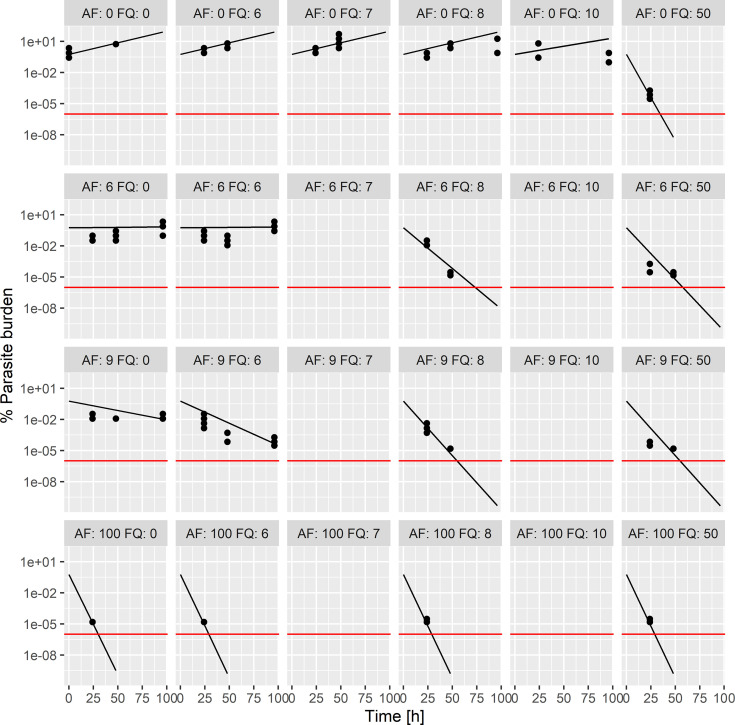
Individual model fits for AF-FQ for the *in vitro* interaction-PRR assay data. Observed parasite burden (black points) and model-predicted parasite burden (black lines) time courses at different concentrations of AF and FQ (numbers denote the concentrations [in nanomolar units] of each agent, covering the EC_20_, EC_80_, and a concentration at the maximum effect). Red lines show quantification limits.

**(ii) AF-FQ combination.** To corroborate the previous results, a second antimalarial combination, with accessible field clinical trial patient data, was studied with the same approach. For the *in vitro* interaction-PRR assay, concentrations of 0, 6, 9, and 100 nM and 0, 6, 7, 8, 10, and 50 nM were chosen for AF and FQ, respectively. Simulations with the PD interaction parameters estimated from the *in vitro* interaction-PRR assay were carried out as described above for the AF-PPQ combination. The parameter estimates of the PK/PD model are presented in Table S3. Assuming Bliss independence, the following PD interactions were estimated: (i) the EC_50_ of FQ was reduced by 68.9% by AF and (ii) the *E*_max_ of AF was reduced by 46.9% by FQ. Hence, the PD interaction was dependent on the concentration. At low concentrations around the EC_50_, a synergistic effect was quantified where the EC_50_ of FQ was reduced by AF. However, at higher concentrations, when *E*_max_ was reached, the *E*_max_ was limited to FQ and the higher *E*_max_ of AF was not reached. This can be seen in Fig. 3, where the slope of AF is steeper at the highest concentration studied in monotherapy than in the combination scenarios. The model predictions agreed well with the observed parasite burden (Fig. 3).

### The PD interactions estimated by the *in vitro* interaction-PRR assay predict parasitological endpoints in humans as well as the estimates obtained with *in vivo* animal model.

**FIG 4:**
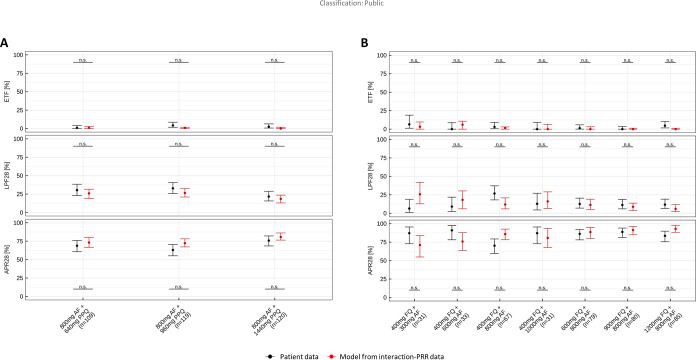
Comparison of parasitological endpoints calculated from observed and predicted parasite burden in the patients receiving (A) AF-PPQ or (B) AF-FQ. The parasitological endpoints—early treatment failure (ETF), late parasitological failure (LPF28), and adequate parasitological response at day 28 (APR28)—are summarized across for all patients per treatment group with AF and PPQ. Error bars depict 90% confidence intervals derived nonparametrically and by the Clopper-Pearson test for simulations and patients’ data, respectively. Black indicates patients’ data, and red indicates simulations with interaction parameters derived from the *in vitro* interaction-PRR assay.

To validate this new assay and the PD model, we used the PD interaction values (on EC_50_ and on *E*_max_) obtained *in vitro* to predict the parasitological endpoints of phase 2 clinical trials. Furthermore, we compared the predictions obtained from our *in vitro* assay with the field clinical trial patient data (Fig. 4A and B). For this purpose, a clinical pharmacometric model was developed that comprised a population pharmacokinetic submodel of the compounds and a pharmacodynamic submodel parameterized with the *E*_max_, EC_50_, Hill factor estimated with clinical monotherapy data, and GPDI interaction estimates stemming from the new *in vitro* assay. The parasitological endpoints adequate parasitological response at day 28 (APR28) and early treatment failure (ETF) calculated from the simulated profiles were compared with those calculated from the observed parasite burden in patients participating in field clinical trials. For both combinations, the *in vitro* interaction-PRR model described the field clinical trials patients’ parasitological endpoints for most dose groups adequately (Fig. 4A and B). The scenarios of AF-PPQ were all well predicted. For AF-FQ, underprediction was observed for APR28 at the lowest doses of AF. However, in none of the scenarios for both drugs was a significant difference between observed and predicted endpoints observed (Fig. 4).

In addition, data from previous studies for AF-PPQ obtained from *in vivo Pfalc*HuEry mouse combination studies were compared to the *in vitro* interaction-PRR assays (Fig. S2; Table S4). We observed that PD interaction parameters derived from the *in vitro* interaction-PRR assay or the *Pfalc*HuEry mouse model both adequately predicted the parasitological endpoints in field clinical trials patients. Of note, the GPDI model parameters from the *Pfalc*HuEry mouse model led to higher uncertainty in the model parameter estimates, likely due to sparser underlying data, and hence, the prediction intervals were larger. Parameter estimates from the *Pfalc*HuEry mouse model are presented in Table S2.

In conclusion, we observed that the *in vitro* interaction-PRR model described the field clinical trial patients’ parasitological endpoints for most dose groups adequately. Furthermore, the *in vitro* interaction-PRR assay allowed us to estimate more complex interaction models, including nonsymmetric interactions with distinct perpetrators and victims within the PD interaction, as well as interactions on the EC_50_ and *E*_max_ levels (Table S4), while the *in vivo* systems allowed estimates only of either EC_50_-based and/or *E*_max_-based interactions and the quantified interaction parameters were mutual. This underlines the value of the richer PD data obtained from the *in vitro* interaction-PRR assay compared to the *in vivo Pfalc*HuEry mouse system.

## DISCUSSION

In the present study, experimental and *in silico*-based approaches were combined to generate informative *in vitro* preclinical data to provide quantitative insights into PD interactions of antimalarial drugs and their translation to the clinical setting. This new *in vitro* combination assay leverages the previously developed gold standard PRR assay (17) to evaluate the maximum parasite killing rate by informing about parasite viability at different time points after drug exposure and makes it possible not only to characterize single-drug effects but also to study drug combinations. The interaction-PRR assay data were quantitatively evaluated using the GPDI model, which provided pharmacological insights into the observed drug interactions, i.e., shifts of EC_50_ and *E*_max_ in comparison to their single-drug effects and the expected additive effect of the combination partners. Moreover, the *in vitro*-derived interaction parameters were used in clinical trial simulations. With the case examples of AF-PPQ, we showed that the *in vitro-*derived parameters led to predictions similar to the interaction parameters derived from the *Pfalc*HuEry mouse *in vivo* model (Fig. S2 and Table S2). This novel *in vitro* assay displays several key properties that advance historically used techniques in preclinical malaria research. Indeed, conventional *in vitro* assays used to study PD interaction solely measure growth inhibition and evaluate if the combined growth inhibition deviates from expected additive growth inhibition, and thus, they do not inform about PD killing interactions. While conventional assays thereby might provide some insight into potency changes in combinations, no information on the potential effects of interactions on parasite killing (i.e., effects on *E*_max_) can be obtained. Another widely used technique to study DDIs is the *in vivo Pfalc*HuEry mouse model. This model can provide quantitative insights into parasite killing, parasite drug resistance (19), and PD drug interactions and thereby also provides valuable information that can be leveraged in a model-based approach for clinical prediction. However, this *in vivo* model (i) is time-consuming, (ii) is very costly, (iii) requires the use of chimeric mice which can pose ethical problems, and (iv) from a modeling perspective provides less rich and informative data than the *in vitro* assay.

The combined experimental-*in silico*-based approach displays several strengths: the development of an *in vitro* assay, in accordance with the 3Rs principle, which provides richer raw data and which is advantageous for exploring the combined response surface as a function of the two drug concentrations in more detail than the previous models. The use of informative concentrations (16) reduces the number of scenarios to be tested to a reasonable number. The required time, workload, and total cost of the *in vitro* PRR assay are far below those of animal experiments. Hence, this novel assay has the potential to streamline the drug development process to select and prioritize combination experiments.

The data generated from the assay are used in conjunction with state-of-the art PK/PD modeling techniques. These models can describe the killing rate of the drugs over time alone and in combination. Thereby, the model not only provides interpretable estimates of the interaction (i.e., shifts of EC_50_ or *E*_max_) but also makes it possible to identify perpetrators and victims of the pharmacodynamic interactions (18). In addition, since this *in vitro*-derived model can describe the parasite killing rate over time, it can be linked to a clinical PK model of the drugs (using either first-in-human or predicted human PK data). This hybrid clinical PK-*in vitro* PD model can then be exploited for clinical trial simulations to evaluate the clinical potential of the combinations by predicting the doses in combination. This will allow the stratification and classification of different antimalarial combination treatments, allowing selection of only those that are efficacious against drug-resistant strains and provide cure within a reasonable time (3 days or less). The approach presented here suggests that animal experiments can be reduced and performed in a confirmatory fashion, thus reducing the number of animals used. However, further research with additional matched case examples will be useful to corroborate our findings.

Some limitations of the study and perspectives for further development are as follows. In contrast to *in vivo* experiments, the interaction-PRR assay does not account for the PK profile of the tested drugs. While this was integrated in the simulations, studying constant concentrations does not provide any insight into persistent drug effects, e.g., an ongoing killing or inhibition of growth after removal of the drug. Moreover, drug degradation could not be included in this study. In future studies, a combination of the interaction-PRR assay with hollow-fiber-type experiments to mimic the PK of single and combination regimens will be evaluated (20, 21). Another interesting use of the interaction PRR assay and the modeling and simulation approach presented here could be to couple the *in vitro*-derived PD model to a *de novo* PK prediction from a physiologically based PK model. Thereby, the combined PK/PD profile could be evaluated before any *in vivo* experiment. Last, although the PRR readout represents the current gold standard to quantify parasite killing, the assay is low throughput, as up to 21 days is needed to detect the regrowth of initial parasites that survived drug exposure. Therefore, the fast PRR assay (22), allowing assessment of parasite viability within a week instead of 21 days, or the use of alternative techniques such as the MitoTracker (23, 24, 25) or immunoenzymatic assays measuring proteins such as Plasmodium falciparum lactate dehydrogenase enzyme or histidine rich protein 2 (26, 27), should be explored to see whether these can provide a comparable but less time-consuming readout to measure parasite killing.

In conclusion, the implementation of this novel alternative translational technology as a routine *in vitro* screening process early in the drug discovery process will facilitate the gathering of more accurate data and improve the quality of preclinical models used to inform first-in-human clinical studies about potential DDIs. With the combination of highly advanced modeling and simulation techniques, the *in vitro*-derived interaction parameters provided predictions similar to those obtained from the more complex *in vivo* mouse model. Moreover, in this study, we showed that this novel *in vitro* assay can provide detailed information about PD interactions of antimalarial drugs, allowing stratification and classification of new antimalarial combinations and potentially in other therapeutic areas. Thus, it helps to optimally select only the most promising combinations and doses for the clinical setting early in the drug development process, which significantly reduces the number of animals conventionally needed in the preclinical phase; it is also hoped that this will minimize the attrition rate in clinical trials.

## MATERIALS AND METHODS

### Monotherapy parasite growth inhibition assay (pretest).

Concentrations to be tested in the interaction-PRR assay are based on inhibitory concentrations in a pretest assay to avoid parasite overgrowth in the PRR step. Briefly, in a 96-well plate, six serial dilutions (1:2) of the desired working dilutions are made. Infected red blood cells (iRBCs; parasitemia, 0.3%; hematocrit, 2.5%) are added on top of the compounds (1:2 dilution; 200-μL final volume per well). Assay plates are incubated at 37°C with 93% N_2_, 4% CO_2_, and 3% O_2_. Parasite growth following drug treatment is quantified via the incorporation of [^3^H]hypoxanthine as previously described (28). After 48 h, a [^3^H]hypoxanthine (0.25 μCi) solution is added to each well, and the plates are incubated for another 24 h. Plates are harvested with a Betaplate cell harvester (Wallac, Zurich, Switzerland), which transfers the lysed red blood cells onto a glass fiber filter. The dried filters are inserted into plastic foil with 10 mL of scintillation fluid and counted in a Betaplate liquid scintillation counter (Wallac, Zurich, Switzerland). The results are recorded as counts per minute per well at each compound concentration. Data are transferred into a graphics program (e.g., Excel or GraphPad Prism) and expressed as percentage of the values for untreated controls.

### Determination of single-drug effects from the growth inhibition assay.

In the first step, the IC_50_ is calculated from conventional growth inhibition data using the sigmoidal maximum effect model with nonlinear regression, relating the observed growth inhibition *I* to the drug concentration *C*:

$$I(C)=\text{baseline}-\frac{I_{\text{max}}\cdot C^{h}}{{\text{IC}}_{50}{}^{h}+C^{h}}$$

where “baseline” represents the observed readout of the growth control, *I*_max_ is the maximum reduction of the readout compared to baseline, IC_50_ is the concentration displaying half-maximal reduction of the readout, and *h* is the Hill coefficient, showing the steepness of the concentration-effect relationship.

The concentrations for the interaction-PRR assay were chosen from the pretest so that at least three informative concentrations were selected covering the IC_20_ (i.e., the concentration stimulating 20% of the of the maximum effect), IC_80_, and a high concentration covering the clinically relevant maximum concentrations, adapted from the work of Chen et al. (16).

### Interaction-PRR assay.

The newly developed *in vitro* interaction-PRR assay is based on the original PRR assay (17). The drug-sensitive P. falciparum strain NF54 was cultivated in RPMI 1640 medium supplemented with 0.5% Albumax II, 25 mM HEPES, 25 mM NaHCO_3_ (pH 7.3), 0.36 mM hypoxanthine, and 100 μg/mL neomycin, as previously described (29, 30). Cultures were maintained in an atmosphere of 3% O_2_, 4% CO_2_, and 93% N_2_ and at 5% hematocrit in humidified modular chambers at 37°C. To initiate the assay, the parasite culture was adjusted to 0.5% parasitemia and 2% hematocrit, and 5 mL of the parasite culture were distributed per well into 6-well plates. Compound powders were dissolved in dimethyl sulfoxide (DMSO) to obtain 10 mM stocks. Thereafter, compounds and compound combinations were prepared in hypoxanthine-free medium and added to the corresponding well to achieve the desired concentrations. An untreated control was included to monitor parasite growth up to 48 h.

Cultures were incubated in an incubation chamber as described above. After 24, 48, 72, and/or 96 h (the sampling schedule was varied depending on the drug to lower the experimental burden), 1 mL of culture was sampled from each well, and the compound was removed by washing twice before resuspending the blood pellet and serially diluting it in 96-well plates. For each time point and each compound or compound combination, 15 serial dilutions of four technical replicates were performed, and afterward, plates were incubated as described above (Fig. S1). Medium was refreshed once a week, and fresh erythrocytes were provided to allow growth of parasites. After 18 to 19 days, the culture medium was removed and replaced with screening medium containing 0.5 μCi of [^3^H]hypoxanthine as previously described (29, 30). Another 48 to 72 h later, plates were frozen at −20°C for a minimum of 24 h. Thawed plates were harvested with a Betaplate cell harvester onto glass fiber filters. The radioactivity was quantified using a Betaplate liquid scintillation counter, and the results were expressed as counts per minute per well. In addition, colored spots on the filter mats were recorded. They served, together with observed medium color changes during the growth period, as visual indicators of parasite growth. Pyrimethamine at 10 times the IC_50_ served as an internal control and was sampled at 24, 48, 72, and 96 h to obtain a full-time-course viability curve. For each technical replicate of a sample, the number of viable parasites was extrapolated using the following equation:

$$P_{\text{viable}}=X^{n-1}$$

where *P*_viable_ represents the number of viable parasites, *X* is the dilution factor used for serial dilution, and *n* is the number of wells exhibiting parasite growth. This number was then used to extrapolate the number of live parasites after treatment in each well.

### Efficacy studies in P. falciparum-infected NSG mice.

*In vivo* preclinical compound efficacy was assessed in the immunodeficient NSG (NOD/SCID/IL2Rγ^−/−^) mouse model engrafted with human erythrocytes and infected with an adapted 3D7 P. falciparum (Pf3D70087/N9) strain (*Pfalc*HuEry mouse model). Briefly, antimalarials were administered alone and/or in combination (AF-PPQ and/or AF-FQ) to a cohort of age-matched female *Pfalc*HuEry mice, as described in the supplemental material.

### Patient efficacy field clinical trials.

The human field clinical trial patient data used in this study were obtained from randomized, single-dose, phase 2b clinical trials as previously described (3, 4). Briefly, confirmed (microscopically) monoinfected field clinical trial patients with P. falciparum were treated with AF and FQ (ClinicalTrials.gov identifier NCT02497612) (3) or AF and PPQ (ClinicalTrials.gov identifier NCT02083380) (4), and parasite burden was monitored by microscopy and/or PCR. The combinations’ efficacy was assessed by the following endpoints: (i) early treatment failure (ETF), i.e., a parasite burden on day 2 posttreatment that was higher than on day 0 or a parasite burden on day 3 that was larger than 25% of the day 0 level; (ii) late parasitological failure at day 28 (LPF28), i.e., parasite burden above the lower limit of quantification (LLOQ) on any day between days 7 and 28 in field clinical trial patients who did not meet the ETF criteria; and (iii) absence of parasite burden at day 28 (APR28), i.e., no parasite burden above LLOQ on day 28 in field clinical trial patients who did not meet the criteria for ETF or LPF28.

### Modelling of pharmacodynamics.

All *in vitro* interaction-PRR assay data were analyzed using nonlinear regression analysis in the NONMEM software (version 7.4; ICON Development Service, Gaithersburg, MD), while assuming constant concentrations.

In the first step, the parasite growth parameters were estimated with an exponential-growth model using viable parasites from the interaction-PRR assay at 48 and 96 h. The growth model is defined as follows:

$$\{\begin{array}{ccc} \frac{\mathrm{dN}}{\mathrm{dt}} & = & k_{\text{growth}}\cdot N\\ N(t=0) & = & N_{0} \end{array}$$

where *N*(*t*) represents the model-predicted viable parasites at time *t*, *k*_growth_ is the first-order growth rate, and *N*_0_ is the initial condition. *N*_0_ was estimated from the back-extrapolated raw readout at 0 h. The raw read (*Y*) was related to *N* by a proportional residual error model and a baseline, which was estimated from the raw readout of the negative controls as follows:

Y = N ⋅ (1 +ε)

Subsequently, the single-drug effects were estimated from the wells containing only single drugs using a sigmoidal maximum-effect model:

$$\frac{\mathrm{dN}}{\mathrm{dt}}=k_{\text{growth}}\cdot N-\frac{E_{\text{max}}\cdot C^{h}}{{\text{EC}}_{50}{}^{h}+C^{h}}\cdot N$$

where *E*_max_ represents the maximum kill rate, EC_50_ is the concentration stimulating half-maximum killing, and *h* is the Hill coefficient, showing the steepness of the concentration-effect relationship.

Thereafter, the combined drug effects for drugs 1 and 2 were evaluated. First, the predicted additivity was computed assuming no drug interaction under Bliss independence criterion fixing of all model parameters. Bliss independence is represented by

$$\frac{\mathrm{dN}}{\mathrm{dt}}=\left(k_{\text{growth}}-E_{1,2}\right)\cdot N$$

where

$$\{\begin{array}{ccc} E_{1} & = & \frac{E_{\text{max},1}\;\cdot \;C_{1}{}^{h_{1}}}{{\text{EC}}_{50,1}{}^{h_{1}}\;+\;C_{1}{}^{h_{1}}}\\ E_{2} & = & \frac{E_{\text{max},2\;}\cdot \;C_{2}{}^{h_{2}}}{{\text{EC}}_{50,2}{}^{h_{2}}\;+\;C_{2}{}^{h_{2}}}\\ E_{1,2} & = & E_{1}+E_{2}-\frac{E_{1}\;\cdot \;E_{2}}{\max\left(E_{\text{max},1},E_{\text{max},2}\right)} \end{array}$$

The model fit is evaluated by computing the Akaike information criterion (AIC). A lower AIC indicates a better model fit for the additivity model, which is then taken forward in the analysis. In case one of the drugs displayed a lag phase, a first-order time delay rate constant $\left(1-e^{-k_{\text{lag}}\cdot t}\right)$ was added on the respective killing rate.

The PD interactions are evaluated using the GPDI model (18) as shifts of *E*_max_ and EC_50_, exemplified here for EC_50_:

$${\text{EC}}_{50,1,\text{GPDI}}={\text{EC}}_{50,1}\cdot \left(1+\frac{{\text{INT}}_{1\leftarrow 2}\cdot C_{2}}{{\text{EC}}_{50,\text{INT},1\leftarrow 2}+C_{2}}\right)$$

$${\text{EC}}_{50,2,\text{GPDI}}={\text{EC}}_{50,2}\cdot \left(1+\frac{{\text{INT}}_{2\leftarrow 1}\cdot C_{1}}{{\text{EC}}_{50,\text{INT},2\leftarrow 1}+C_{1}}\right)$$

where EC_50,1,GPDI_ represents the EC_50_ of drug 1 being shifted by drug 2 at maximum by INT_1←2_ at a potency of EC_50,INT,1←2_, and vice versa for EC_50,2,GPDI_. The GPDI model is built up using statistical criteria (likelihood ratio test, α = 0.05, *df* = 1; all model parameters estimated) starting with a reduced model with a single INT parameter for each drug indicating monodirectional interactions, up to estimating both INT parameters and finally also estimating the interaction potencies (EC_50,INT,_*_i_*_←_*_j_*).

An estimated INT parameter between −1 and 0 indicates that the respective EC_50_ is decreased in the presence of the other drug, demonstrating synergy. An estimated INT parameter not significantly different from zero indicates additivity. An estimated INT parameter of >0 indicates that the EC_50_ is increased in the presence of the other drug, demonstrating antagonism. INT parameters of opposite polarity indicate asymmetric interactions where overall antagonism or synergy cannot be concluded, and interactions are concentration dependent.

### Clinical trial simulations and comparison using *Pfalc*HuEry mouse and *in vitro* interaction-PRR assay estimates.

**FIG 5:**
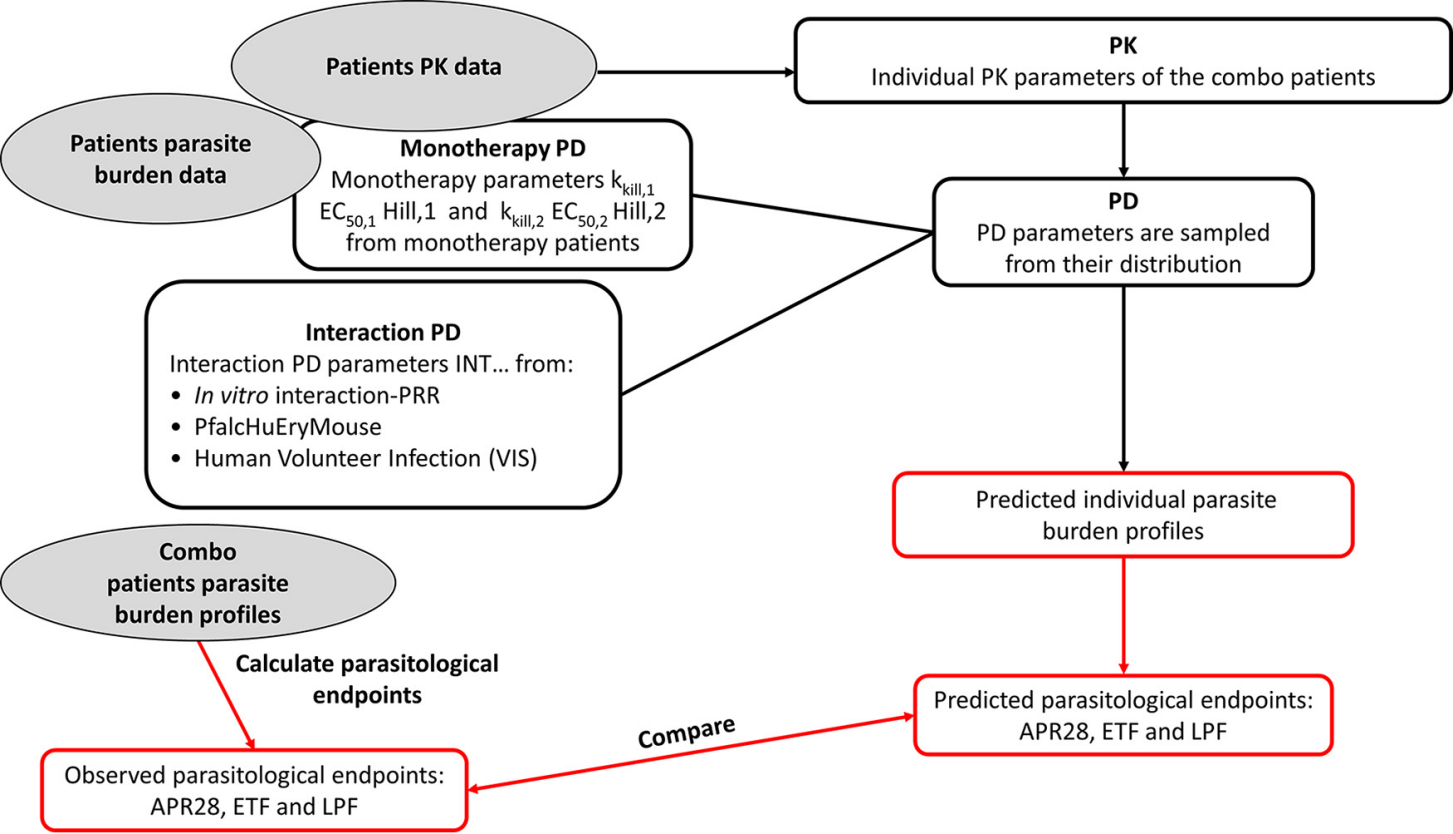
Simulations of clinical trials. Simulations of clinical combination trials are run with individual PK parameters estimated with the PK concentrations of the combo trial, the monotherapy PD parameters estimated from clinical monotherapy trials, and the interaction PD parameters estimated either from *in vitro* interaction-PRR or the PfalcHuEry mouse model. The individual parasite burden profiles are simulated with this model. The parasitological endpoints (APR28, ETF, and LPF) are calculated from these as well as from the observed parasite burden profiles. The predicted and observed endpoints thus obtained are then compared.

The ability to predict the outcome of combination therapies in humans (clinical trials) using the *Pfalc*HuEry mouse model and *in vitro* interaction-PRR parameter estimates was assessed in clinical trial simulations with two antimalarial combination treatments. Treatment effects in individual patients were simulated using interaction parameters from an *in vitro* interaction-PRR assay and *Pfalc*HuEry mouse model experiments and compared to field clinical study data, as depicted in the workflow in Fig. 5. For each subject in the clinical trial, parasite burden was simulated using individual PK parameters, baseline parasite burden (PL_base_) and parasite growth rates, and respective monotherapy PK/PD models and drug PD interaction estimates from the *Pfalc*HuEry mouse model or *in vitro* interaction-PRR. The clinical trials were simulated 250 times with PD parameters sampled from uncertainty, resulting in 250 simulated trials. For each simulated trial, interaction parameters were sampled from uncertainty, described by the relative standard error, based on estimation results from *in vitro* interaction-PRR and/or the *Pfalc*HuEry mouse model. Furthermore, for each trial, *E*_max_, EC_50_, *h*, and the standard deviation (SD) for interindividual variability (IIV) were sampled from uncertainty. Individual values for *E*_max_, EC_50_, and *h* were then drawn from IIV for each individual within a trial. The parasite burden profile was calculated from the PK/PD model for each individual. A cure threshold of 1 parasite per body, which corresponds to 1 parasite per 70 mL of blood per kg of body weight was defined. Once parasite burden reached this threshold, it was annotated as “cured” for the remaining simulation time, to indicate a complete elimination of parasites. Then, for each subject, the endpoints ETF, LPF28, and APR28 were calculated.

The fraction of individuals achieving the simulated parasitological endpoint was calculated for each dose group within each simulated trial. Then, median and 90% confidence interval (CI) were computed for each dose group across the 250 simulated trials. Simulations and field clinical trial patient data were compared using the Kolmogorov-Smirnov test. Due to the binary output for the endpoints, the fractions of individuals achieving an endpoint per trial were discrete. For statistical tests, the output of the simulations was smoothened using the bandwidth of the kernel density estimate. Resulting negative values of this smoothening procedure were set to 0. *P* values were adjusted for multiple testing using the Benjamini-Hochberg procedure for each model (31).

### Data availability.

The data sets analyzed during the current study are available from the corresponding author on request.
