# Supplementary material for: New In Vitro Interaction-Parasite Reduction Ratio Assay for Early Derisk in Clinical Development of Antimalarial Combinations
Source: Antimicrob Agents Chemother. 2022 Oct 5;66(11):e00556-22. doi: 10.1128/aac.00556-22 (PMC9664866; doi:10.1128/aac.00556-22)
Supplement: Supplemental file 1 — Supplemental material. Download aac.00556-22-s0001.pdf, PDF file, 0.5 MB [file aac.00556-22-s0001.pdf]

Supplementary Information

A new in vitro **interaction-parasite reduction ratio assay for early de-risk of clinical development of antimalarial combinations**

Sebastian G. Wicha[1*], Annabelle Walz[2,3], Mohammed H. Cherkaoui-Rbati[4], Nils Bundgaard[5], Karsten Kuritz[5], Christin Gumpp[2,3], Nathalie Gobeau[4], Jörg Möhrle[4], Matthias Rottmann[2,3], Claudia Demarta-Gatsi[4#*]

Affiliations

[1] Department of Clinical Pharmacy, Institute of Pharmacy, University of Hamburg, Bundesstr. 45, 20146 Hamburg, Germany

[2] Department of Medical Parasitology and Infection Biology, Swiss Tropical and Public Health Institute, Kreuzstr. 2, CH-4123 Allschwil, Switzerland

[3] University of Basel, Basel, Switzerland

[4] Medicines for Malaria Venture, Geneva, Switzerland

[5] IntiQuan GmbH, Elisabethenstr. 23, 4051 Basel, Switzerland

[#] Current address: Global Health Institute of Merck, Ares Trading S.A. (a subsidiary of Merck KGaA Darmstadt Germany), 1262 Eysins, Switzerland

[*] Correspondence and requests for materials should be addressed to CDG or SW (email: claudia.demarta@external.merckgroup.com, sebastian.wicha@uni-hamburg.de).

Supplementary Materials and Methods

Efficacy studies in *P. falciparum*-infected NSG mice

Compound efficacy was assessed in the immunodeficient NSG (NOD.Cg-Prkdc*scid*Il2rgtm1WjI/SzJ) mouse model engrafted with human erythrocytes and infected with an adapted *P. falciparum* (Pf3D70087/N9) strain (*Pfalc*HuEryMouse model) as described by Jimenez-Díaz *et al. (1)*. Briefly, antimalarials were administered alone and/or in combination (AF-PPQ and/or AF-FQ) to a cohort of age matched female *Pfalc*HuEryMice. Drug treatments were performed on day 3 after infection; mice were administered with oral gavage in a vehicle volume of 10 mL/kg bodyweight, at different doses Table below.The quantification of parasitaemia at different timepoints after the infection was performed by flow cytometry as previously described *(2)*. The blood concentrations of the different compounds in mice were measured in serial samples of peripheral blood taken by tail puncture at different time points after treatment. The blood samples were immediately lysed and stored at −80°C until analysis by liquid chromatography–tandem mass spectrometry. The analysis was conducted at the Art of Discovery (TAD), Spain, and approved by The Art of Discovery Institutional Animal Care and Use Committee (TAD-IACUC). This committee is certified by the Biscay County Government (Bizkaiko Foru Aldundia, Basque Country, Spain) to evaluate animal research projects from Spanish institutions according to point 43.3 from Royal Decree 53/2013, from the 1st of February (BOE-A-2013-1337).

Compound	1 x Dose (mg/kg)	2 x Dose (mg/kg)	3 x Dose (mg/kg)	4 x Dose (mg/kg)
AF	15, 50 and 100	15	-	15
PPQ	10, 20 and 40	5 and 20	5	-
FQ	7.5, 10 and 15	-	-	-

Modelling of PK and PD of mouse data

In mice experiments a PK/PD model was built to identify the PD interactions. Monotherapy data from previous clinical trials were added to the combination dataset. The modelling was done in two stages: first, a PK model was built to obtain individual PK parameter estimates; second, these parameters were used as regressors to calculate the individual time-concentrations and the PD parameters were estimated.

Modelling of PK

In field clinical trials, a PK model was built, and the individual PK parameters were used in the simulations. In all analyses, the R package IQRtools (https://iqrtools.intiquan.com/) was used. Mice PK models were developed with the IQRtools Sysfit approach *(3)*. When few subjects were available and estimation of the variance of random effects was difficult, the Sysfit approach was preferred to the NLME method. To build the PK model, first a separate PK model for each compound was built: one, two, three-compartment models were tested with different absorption models (first order; with and without lag phase) and different clearance models (linear or saturable). The best model was selected based on Akaike Information Criterion (AIC) and goodness-of-fit plots. A PK model of a combination was then built by combining the separate PK models of the two compounds and adding a PK interaction term where the clearance of one drug is affected by the concentration of the other drug. The term was kept if it was found statistically significant.

Modelling of PD

Modelling of PD of mouse data was performed similar as described in the main part of the manuscript. Mice experiments were analysed with the R package IQRtools using the Sysfit approach. The individual PK parameters obtained previously were used as regressors to compute the individual time-dependent concentrations.

Supplementary Figures

Figure S1: Schematic presentation of the assay procedure. Parasites (0.5% parasitaemia, 2% haematocrit) are incubated in 6-well plates for 24, 48, 72 and 96 hours. Compound was removed by washing. Four technical replicates of washed sample were then serially diluted by factor three over a range of sixteen wells of a 96-well plate. Parasite growth was monitored after 21 days by [^{3}H] hypoxanthine readout. A restriction in the PRR assay is, that only a limited number of concentrations be tested. Hence, optimised concentrations were chosen from a previously conducted growth inhibition experiment and comprise the EC20, EC50 and EC80 and 10x EC50 which are informative to capture pharmacodynamic drug interactions *(4)*. The concentration levels were then combined in a checkerboard fashion *(5)*.

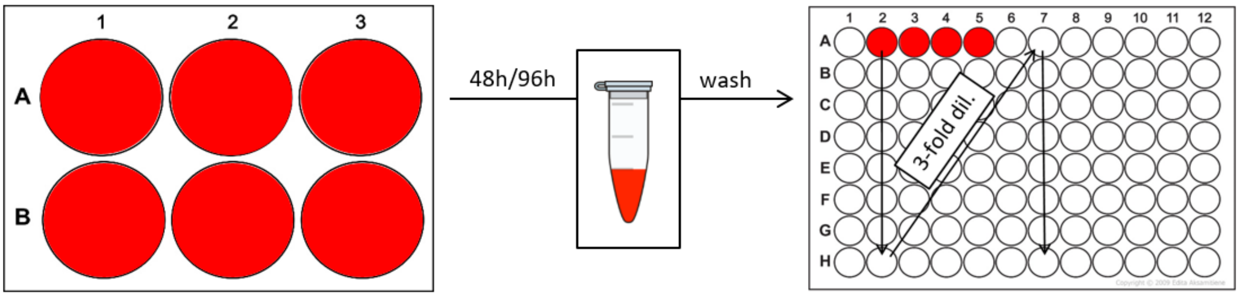

Figure S2: Comparison of parasitological endpoints calculated from observed and predicted parasite burden in field clinical trial patients receiving AF-PPQ combination. The parasitological endpoints: early treatment failure (ETF), late parasitological failure at day 28 (LPF28) and adequate parasitological response at day 28 (APR28) are summarized across for all field clinical trial patients per treatment group with of AF and PPQ. Error bars depict 90% confidence intervals derived non-parametrically and by Clopper-Pearson for simulations and field clinical trial patients' data respectively. Colour codes for field clinical trial patients' data (black), and simulations with interaction parameters derived from *in vitro* interaction-PRR (red) and *Pfalc*HuEryMouse (blue). Kolmogorov-Smirnov test was used for statistical analysis.

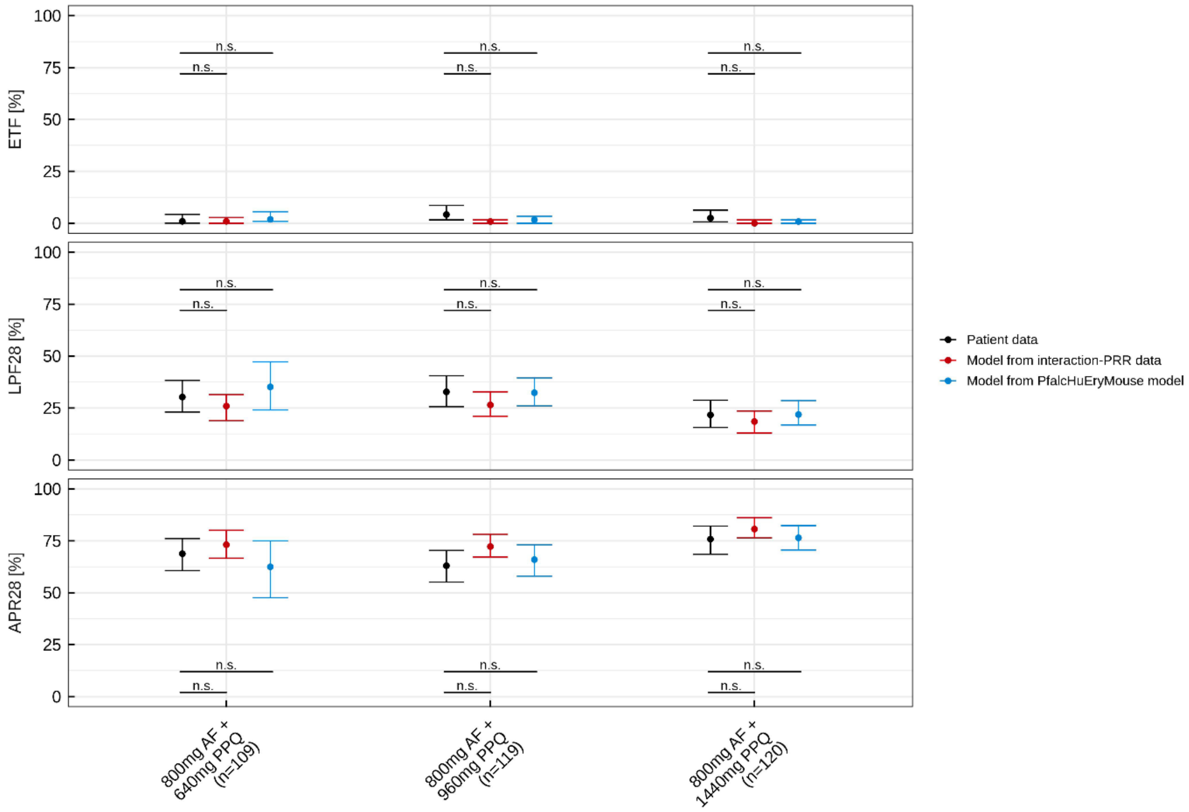

Supplementary Tables

Table S1: Parameter estimates of the PKPD model describing the *in vitro* interaction-PRR experiment between artefenomel (AF) and piperaquine (PPQ).

Parameter	Explanation	Estimate	Rel. standard error [%]	CI 95 %
N0 [%]	% parasite burden at t=0 h	0.232	Fixed to experimental value	-
KG [/h]	Growth rate constant	0.133	5	0.122; 0.146
Emax,1 [/h]	Maximum kill rate AF	0.406	3	0.382; 0.429
EC50,1 [nM]	Conc. stimulating 50% of Emax	12.7	4	11.8; 13.7
Hill,1 [-]	Steepness of the conc.-effect relationship	1.68	7	1.46; 1.88
Emax,2 [/h]	Maximum kill rate PPQ	0.267	3	0.254; 0.280
EC50,2 [nM]	Conc. stimulating 50% of Emax	9.34	2	9.07; 9.63
Hill,2 [-]	Steepness of the conc.-effect relationship	7.99	7	6.89; 9.13
$INT_{12,EC50}$ [-]	Maximum fractional change of EC50 of AF mediated by PPQ	10.7	44	5.74; 20.1
$INT_{21,EC50}$ [-]	Maximum fractional change of EC50 of PPQ mediated by AF	-0.355	9	-0.416; -0.301
$INT_{12,Emax}$ [-]	Maximum fractional change of Emax of AF mediated by PPQ	-0.14	20	-0.189; -0.090
$INT_{21,Emax}$ [-]	Maximum fractional change of Emax of PPQ mediated by AF	0.316	9	0.257; 0.373
$EC50_{INT12,EC50}$ [nM]	Potency of the EC50 interaction of AF mediated by PPQ	4.9	14	3.63; 6.34
$EC50_{INT21,EC50}$ [nM]	Potency of the EC50 interaction of PPQ mediated by AF	= $EC50_{INT12,EC50}$	-	
$EC50_{INT12,Emax}$ [nM]	Potency of the Emax interaction of AF mediated by PPQ	= EC50,2	-	
$EC50_{INT21,Emax}$ [nM]	Potency of the Emax interaction of PPQ mediated by AF	= EC50,1	-	
Residual prop. error [%CV]		97.7%	31	39.1223; 160.278

Table S2: Parameter estimates of the PKPD model describing the *in vivo* PfalcHuEryMouse experiment between artefenomel (AF) and piperaquine (PPQ).

**Parameter**	**Explanation**	**Estimate**	**Rel. standard error [%]**	**CI 95 %**
KG [/h]	Growth rate constant	0.064763	Fixed	-
Emax,1 [/h]	Maximum kill rate AF	0.2179	3.75	0.202; 0.234
EC50,1 [ug/mL]	Conc. stimulating 50% of Emax	0.0384	7.17	0.033; 0.044
Hill,1 [-]	Steepness of the conc.-effect relationship	3.2	17.86	2.08; 4.32
Emax,2 [/h]	Maximum kill rate PPQ	0.265	2.84	0.25; 0.28
EC50,2 [nM]	Conc. stimulating 50% of Emax	0.0085	5.9	0.008; 0.009
Hill,2 [-]	Steepness of the conc.-effect relationship	5.56	12.48	4.2; 6.92
INT_{EC50} [-]	Maximum fractional change of EC50	5.06	7.68	4.298; 5.822
INT_{Emax} [-]	Maximum fractional change of Emax	0	0	-
$EC50_{INT12,EC50}$ [nM]	Potency of the EC50 interaction of AF mediated by PPQ	0.021492	0	-
$EC50_{INT21,EC50}$ [nM]	Potency of the EC50 interaction of PPQ mediated by AF	0.0415	0	-
$EC50_{INT12,Emax}$ [nM]	Potency of the Emax interaction of AF mediated by PPQ	0.021492	0	-
$EC50_{INT21,Emax}$ [nM]	Potency of the Emax interaction of PPQ mediated by AF	0.0415	0	-
Residual prop. error		1.219	3.948	0.2757; 2.162

Table S3: Parameter estimates of the PKPD model describing the *in vitro* interaction-PRR experiment between artefenomel (AF) and ferroquine (FQ).

**Parameter**	**Explanation**	**Estimate**	**Rel. standard error [%]**	**CI 95 %**
N0 [%]	% parasite burden at t=0 h	0.57	Fixed to experimental value	
KG [/h]	Growth rate constant	0.053	6	0.047; 0.059
Emax,1 [/h]	Maximum kill rate AF	0.529	3	0.505; 0.565
EC50,1 [nM]	Conc. stimulating 50% of Emax	21.4	12	18.8; 26.1
Hill,1 [-]	Steepness of the conc.-effect relationship	1.77	11	1.51; 2.03
Emax,2 [/h]	Maximum kill rate FQ	0.436	2	0.414; 0.452
EC50,2 [nM]	Conc. stimulating 50% of Emax	11.4	8	10.8; 12.5
Hill,2 [-]	Steepness of the conc.-effect relationship	24.9	62	17.1; 37.5
$INT_{21,EC50}$ [-]	Maximum fractional change of EC50 of FQ mediated by AF	-0.689	32	-0.704; -0.570
$EC50_{INT,21,EC50}$ [nM]	Potency of AF in the EC50 interaction of FQ mediated by AF	6.22	61	4.27; 10.8
$INT_{12,Emax}$ [-]	Maximum fractional change of Emax of FQ mediated by AF	-0.469	9	-0.575; -0.405
$EC50_{INT,12,EMAX}$ [nM]	Potency of AF in the Emax interaction of FQ mediated by AF	1.96	51	5.4E-05; 3.68
Residual prop. error [%CV]		133.7	22	76.0486; 191.351

Table S4: Artefenomel - piperaquine interactions parameter estimates of PKPD models and comparison of the *in vitro* interaction-PRR and *Pfalc*HuEryMouse model.

Case study	Interaction parameters	*In vitro*	*Pfalc*HuEryMouse
Artefenomel Piperaquine	Maximum fractional change of E_{max} of Artefenomel mediated by partner	-0.14 [-0.189; -0.090]	0 Fixed
	Maximum fractional change of E_{max} of partner mediated by Artefenomel	0.316 [0.257; 0.373]	0 Fixed
	Maximum fractional change of EC_{50} of Artefenomel mediated by partner	10.7 [5.74; 20.1]	5.06 [4.29; 5.82]
	Maximum fractional change of EC_{50} of partner mediated by Artefenomel	-0.355 [-0.416; -0.30]	5.05 [4.29; 5.82]

BIBLIOGRAPHY

1. M. B. Jiménez-Díaz, T. Mulet, S. Viera, V. Gómez, H. Garuti, J. Ibáñez, A. Alvarez-Doval, L. D. Shultz, A. Martínez, D. Gargallo-Viola, I. Angulo-Barturen, Improved murine model of malaria using Plasmodium falciparum competent strains and non-myelodepleted NOD-scid IL2Rgammanull mice engrafted with human erythrocytes. *Antimicrob. Agents Chemother.* **53**, 4533–4536 (2009).

2. M. B. Jiménez-Díaz, T. Mulet, V. Gómez, S. Viera, A. Alvarez, H. Garuti, Y. Vázquez, A. Fernández, J. Ibáñez, M. Jiménez, D. Gargallo-Viola, I. Angulo-Barturen, Quantitative measurement of Plasmodium-infected erythrocytes in murine models of malaria by flow cytometry using bidimensional assessment of SYTO-16 fluorescence. *Cytometry. A* **75**, 225–35 (2009).

3. D. Kaschek, W. Mader, M. Fehling-Kaschek, M. Rosenblatt, J. Timmer, Dynamic Modeling, Parameter Estimation, and Uncertainty Analysis in R. *J. Stat. Softw.* **88**, 1–32 (2019).

4. C. Chen, S. G. Wicha, G. J. de Knegt, F. Ortega, L. Alameda, V. Sousa, J. E. M. de Steenwinkel, U. S. H. Simonsson, Assessing Pharmacodynamic Interactions in Mice Using the Multistate Tuberculosis Pharmacometric and General Pharmacodynamic Interaction Models. *CPT pharmacometrics Syst. Pharmacol.* **6**, 787–797 (2017).

5. S. G. Wicha, M. G. Kees, J. Kuss, C. Kloft, Pharmacodynamic and response surface analysis of linezolid or vancomycin combined with meropenem against Staphylococcus aureus. *Pharm. Res.* **32**, 2410–2418 (2015).
